# Dispersibility and Size Control of Silver Nanoparticles with Anti-Algal Potential Based on Coupling Effects of Polyvinylpyrrolidone and Sodium Tripolyphosphate

**DOI:** 10.3390/nano10061042

**Published:** 2020-05-29

**Authors:** Mingshuai Wang, Haibo Li, Yinghua Li, Fan Mo, Zhe Li, Rui Chai, Hongxuan Wang

**Affiliations:** School of Resources and Civil Engineering, Northeastern University, Shenyang 110819, China; wms1995@126.com (M.W.); moliangming4312975@163.com (F.M.); lizhe1824@163.com (Z.L.); chairuicici@126.com (R.C.); 18245706557@163.com (H.W.)

**Keywords:** silver nanoparticles, dispersibility, coupling mechanism, anti-algal

## Abstract

In nearly all the cases of biotoxicity studies of silver nanoparticles (AgNPs), AgNPs used often have general dispersibility and wide size distribution, which may inevitably generate imprecise results. Herein, a kind of synthesis method by coupling effects of polyvinylpyrrolidone (PVP) and sodium tripolyphosphate (STPP) was proposed, in order to prepare AgNPs with better dispersibility and a stable size. Based on this, the preparation mechanism of AgNPs and the potential anti-algae toxicity were analyzed. UV-vis analysis showed that the particle size distribution of AgNPs prepared by co-protective agents was more uniform. X-ray diffraction (XRD), field emission scanning electron microscopy (FE-SEM), transmission electron microscopy (TEM), and energy dispersive X-ray (EDX) were used to confirm that the obtained nano silver was of a high purity and stable size (~30 nm in diameter). Zeta potential and Fourier transform infrared spectroscopy (FTIR) analysis results indicated the synthesis mechanism of AgNPs by co-protective agents, more precisely, PVP limited the polynegative effect and prevented the linear induction of P_3_O_10_^5−^ produced by STPP during the growth of silver nuclei. Subsequently, *Chlorella* and *Scenedesmus obliquus* were utilized to test the toxicity of AgNPs, confirming that AgNPs synthesized through co-protective agents have potential inhibitory ability on algae, but not severe. This study provides a basic theory for the induction of synthetic AgNPs by various factors in the natural environment and a scientific reference for the environmental risk assessment.

## 1. Introduction

Over the past decades, silver nanoparticles (AgNPs) have been widely applied for their unique dimensional structures and performance superiorities, such as antibacterial [1,2], antifungal [3,4], anti-algal [5,6], catalytic [7,8], and photoelectric properties [9,10]. Many investigations into the biological and chemical synthesis methods of AgNPs with multiple shapes and sizes have been reported [11,12,13,14,15]. Although these previous results were expansive, data to explain the growth process are still scare. Such an explanation requires the exploration and perfection of the synthetic mechanism.

In general, there are two protective mechanisms for maintaining the stability of nanoparticles during chemical synthesis, namely the electrostatic repulsion of small molecule protectants [16] and the steric hindrance of macromolecular polymers [17]. When the pH of an aqueous solution is higher than the zero-potential pH (pH_PZC_) of nano silver, its surface without coating adsorbs some negatively charged groups (e.g., hydroxide ions and oxygen-containing groups) and maintains stability via electrostatic repulsion [18]. Nano silver that is stabilized by steric hindrance has a stronger anti-electrolyte interference ability and can exist stably under relatively high ionic-strength conditions [19]. However, few studies [20,21] have combined two protective mechanisms to explore their common mechanism for the synthesis of AgNPs.

Furthermore, in the biotoxicity experiments of AgNPs, the studies show that the most obvious toxic effect of AgNPs on algae is to inhibit growth [22]. It may also adsorb and aggregate on the surface of algae cells, which in turn leads to the destruction of the cell membrane or skeleton structure and to the damage of organelle functions, thus inhibiting the material metabolism and photosynthesis of algal cells [23]. However, most of them used AgNPs with general dispersibility and wide particle size distribution for experiments, which would affect the evaluation of their biological toxicity and make them difficult to be convinced. The dispersibility of AgNPs in the water environment may have a difference in the toxicity of biomolecules, and this difference has not been confirmed.

Therefore, a hypothesis would be proposed that sodium tripolyphosphate (STPP) was used as a small molecule protectant and polyvinylpyrrolidone (PVP) was used as a macromolecular polymer to investigate whether coupling mechanism would occur during the synthesis of AgNPs. The steric hindrance effect of PVP and the electrostatic repulsion effect of STPP may synergistically promote the dispersibility and stability of AgNPs or affect the entire redox process. In this present study, PVP and STPP were used together as a protective agent for synthesizing AgNPs, with silver nitrate (AgNO_3_) and glucose as the precursor of silver and the reducing agent, respectively. The obtained nanoparticles were characterized by ultraviolet-visible (UV-vis) spectroscopy, X-ray diffraction (XRD), field emission scanning electron microscopy (FE-SEM), transmission electron microscopy (TEM), zeta potential, and Fourier transform infrared spectroscopy (FTIR) analyses, as a means of explaining the coupling mechanism of particle size growth and stability during the synthesis process. Finally, *Chlorella* and *Scenedesmus obliquus* were selected to compare the toxicity of nano silver, and we examined the inhibition potential of AgNPs with different dispersibility to algae.

## 2. Materials and Methods

### 2.1. Materials

Silver nitrate (AgNO_3_) (99.8%) and sodium tripolyphosphate (Na_5_P_3_O_10_), which were both analytical reagents (ARs), were supplied by Sinopharm Chemical Reagent Co. Ltd. (Shenyang, China) Polyvinylpyrrolidone (PVP) (K30) was obtained from Macklin (Shanghai, China). Glucose and sodium hydroxide (Zhiyuan Chemical Reagent Co. Ltd., Tianjin, China) were both reagent-grade, as-received materials. Deionized water was used as a solvent throughout the experiment.

### 2.2. Preparation of AgNPs

Several reagent powders were dissolved in deionized water, using glucose (0.1 mol/L) as the reducing solution, AgNO_3_ (0.01–0.05 mol/L) as the precursor of silver, PVP (0.1 mol/L), and STPP (0.05 mol/L) as two protective solutions. The reducing solution, protective solutions, and sodium hydroxide (0.05 mol/L) were mixed in a three-necked flask. In a constant temperature water bath, the solution was stirred for 5 min, to even the mixture. Afterward, we added AgNO_3_ solution, dropwise, into the flask. Nano silver was obtained after continued heating (bath heating at 80 °C) and stirring, and was then purified by multiple water washings and high-speed centrifugations. Detailed parameters for preparing AgNPs are presented in Tables S1–S3.

### 2.3. Characterization of AgNPs

#### 2.3.1. Ultraviolet-Visible (UV-Vis) Spectroscopy

The synthesis of AgNPs was confirmed through UV-vis spectroscopy, at 350 to 600 nm, on a UV-vis spectrophotometer (Hach DR6000, Loveland, CO, USA). The mother liquor containing AgNPs was diluted thirty times, to avoid noise signals, and deionized water was taken as reference.

#### 2.3.2. X-Ray Diffraction (XRD)

The XRD (Panaco X Pertpro, Almelo, The Netherlands) technique was an effective method to identify the crystal phase of substances. The average particle size in the direction of the corresponding crystal plane was calculated by using the Scherrer equation. The XRD diffraction intensities were recorded from 20° to 90° in 2θ angles, and patterns were analyzed through X’ Pert High Score Plus software (PANalytical B.V., Almelo, The Netherlands).

#### 2.3.3. Field Emission Scanning Electron Microscopy (FE-SEM)

The size and morphology of nano silver were evaluated by FE-SEM (Hitachi S-4800II, Tokyo, Japan). The test samples were obtained via the drop-casting method, in which a small amount of AgNPs dispersed in 95% alcohol was first dropped onto a silicon chip and then dried at room temperature, to deposit. Later, the silicon chip was examined at an acceleration voltage of 2 kV. The obtained FE-SEM images of nano silver were subsequently processed for their histograms by Nano Measurer 1.2 software (Fudan University, Shanghai, China). Elemental analysis of nanoparticles was performed by using the equipped energy dispersive X-ray (EDX, Hitachi Limited, Tokyo, Japan).

#### 2.3.4. Transmission Electron Microscopy (TEM)

The morphology and dispersion of nano silver were further authenticated by TEM (Hitachi HT7700, Tokyo, Japan), operated at an accelerating voltage of 120 kV. A drop of colloidal nanoparticles was placed on a 300-mesh copper grid with a carbon film support and dried for 24 h prior to observation.

#### 2.3.5. Zeta Potential

Zeta potential is the scale of electrostatic interaction in nanoparticles and can be used to predict the stability of the dispersion system. The zeta potential of AgNPs was determined with a Malvern Zetasizer NanoZS instrument (Malvern Panalytical Ltd., Malvern, UK).

#### 2.3.6. Fourier Transform Infrared Spectroscopy (FTIR)

FTIR (Seymour Nicolet iS10, Waltham, MA, USA) identified different types of functional groups in particles. Measurements were carried out, using scanning wavelengths of 600–4000 cm^−1^, with a resolution of 0.4 cm^−1^. The obtained peaks were compared with standard functional group charts.

### 2.4. Anti-Algal Activity of AgNPs

#### 2.4.1. Algae Cultivation

We selected *Chlorella* and *Scenedesmus obliquus* as experimental subjects, which were unicellular green eukaryotic microalga. Both algae are outstanding single-cell green eukaryotic microalgae. They can easily grow in freshwater and have a short culture period, which is preferred for biological assays. Their cell structure is simple, and the chloroplast contains the green photosynthetic pigments chlorophyll a and b. It is believed to have high potential for photosynthesis efficiency. We purchased two algae species from Freshwater Algae Culture Collection at the Institute of Hydrobiology, Wuhan, China, and cultured them in BG11 medium. An artificial climate equipment was used to continuously provide a temperature control of 25 °C and 2000 lux light intensity. The growth and cultivation status of algae are available in the Figures S1 and S2.

#### 2.4.2. Photosynthetic Pigment Measurement

In order to test the photosynthetic pigments, different concentrations of AgNPs (0.1, 0.3, and 0.5 mmol/L) were added to the cultured algal solution. Two parallel experiments were conducted. The extraction method of photosynthetic pigment was improved based on the method proposed by Sartory and Grobbelaar [24]. On the fifth day of incubation, 5 mL of algal culture solution was filtered through a 0.45 µm filter membrane, which then was cut into pieces and put into a centrifuge tube. After using alcohol (95%) for extraction, it was dispersed in ultrasound for 15 min, followed by centrifugation at 8000 rpm for 8 min. We collected the supernatant and measured the absorbance at 645 and 663 nm, using a UV-vis spectrophotometer. The total chlorophyll content was calculated according to the Arnon formula (Equation (1)) [25].

Total chlorophyll (mg/g) = ([(OD_645_ × 20.2) + (OD_663_ × 8)] × *V*)/(1000 × *W*) (1)

where *V* is the chlorophyll extraction volume (mL), and *W* is the biomass weight of algal (g).

## 3. Results and Discussion

### 3.1. Synthesis and Parameter Control

#### 3.1.1. Individual Protective Agents versus Combined Protective Agents

**Figure 1:**
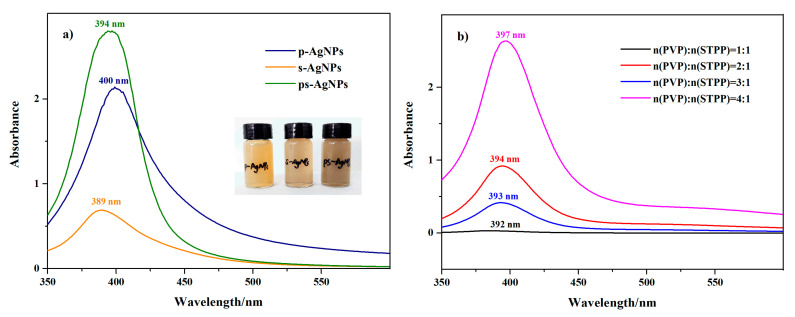
(**a**) Ultraviolet-visible (UV-vis) spectra of individual protective agents versus combined protective agents. (**b**) UV-vis spectra of silver nanoparticles (AgNPs) with different initial concentrations of AgNO_3_. Polyvinylpyrrolidone is indicated by p-, and sodium tripolyphosphate by s-.

AgNPs have a high surface energy and are extremely unstable under the condition of no protective agent coating. Adding a protective agent to the liquid-phase system to reduce the energy of nano silver has a great influence on its size and structural formation [26,27]. Three AgNPs (p-AgNPs, s-AgNPs, and ps-AgNPs) were prepared by Ag/glucose colloidal solution, under different protective agents (PVP, STPP, and PVP plus STPP), which exhibited surface plasmon absorption band peaks of 400, 389, and 394 nm, respectively (Figure 1a). Compared with the individual protective agents, the position of the surface plasmon absorption peak of the AgNPs synthesized by the two protective agents was intermediate, which means that the particle size of ps-AgNPs was between p-AgNPs and s-AgNPs. Additionally, the colloidal concentration of ps-AgNPs was larger, and the size distribution was relatively uniform by analyzing the peak height and width. After a period of time, a large amount of gray–black precipitate appeared in the colloids prepared by STPP as a protective agent, while the other two kinds of colloids basically remained in a dispersed state. This phenomenon indicated that the nano silver colloids prepared by STPP were very unstable. The s-AgNPs were rapidly agglomerated, i.e., its nanoparticles that were of a broad size range were formed with the passing of time [28].

The effect of the molar ratio of protective agent was investigated on the formation of nanoparticles, under constant conditions (0.02 M AgNO_3_, 15 mL glucose and bath heating at 80 °C), as shown in Figure 1b. Due to the strong surface plasmon resonance (SPR), AgNPs absorbed approximately 394 nm of visible radiation. The comparison results illustrated that, when the ratio of PVP/STPP was 4:1, a higher and narrower absorption peak of the waveform was obtained. Therefore, the synergistic effect of the electrostatic repulsion and steric hindrance was easily affected by the proportion of co-protective agents. After the ratio of protective agent was changed, the characteristic SPR band did not exhibit any red or blue shift, thus suggesting that the particle size was stable substantially. Hence, an appropriate adjustment of the ratio of the protective agent was beneficial to obtain a regular AgNPs with a uniform size, which presented better stability and dispersion of AgNPs when the steric hindrance of PVP dominated.

#### 3.1.2. Effects of Temperature and Silver Nitrate Concentration

**Figure 2:**
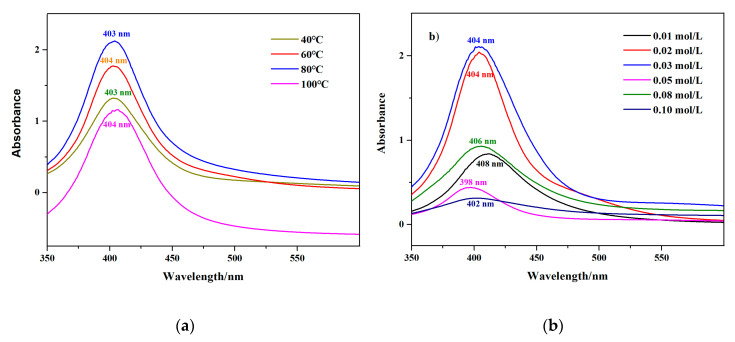
(**a**) Ultraviolet-visible (UV-vis) spectra of ps-AgNPs synthesized at different temperatures, using 0.02 mol/L AgNO_3_. (**b**) UV-vis spectra of ps-AgNPs synthesized with different initial concentrations of AgNO_3_.

Figure 2a displays the UV-vis absorption spectra of AgNPs prepared at different temperatures (40, 60, 80, and 100 °C), with a reaction time of 20 min. At first, as the temperature rose from 40 to 80 °C, the absorption peak at 400 nm gradually became stronger; hence, more silver ions were converted into nano silver. In addition, such a raise in temperature would have accelerated the movement of ions, thereby making electrostatic forces active, which would have enhanced the stability. However, a further increase to 100 °C would have led to a decrease in the maximum absorbance value. This might indicate that the higher temperatures accelerated the rate of AgNPs synthesis, thus causing the faster nucleation growth. When the particle size of nano silver became larger, its dispersibility deteriorated, resulting in agglomeration [29]. Therefore, an equilibrium point between a good synthesis rate and a uniform particle size distribution were found at the reaction temperature of 80 °C. An even-higher temperature might destroy the electrostatic repulsion between AgNPs.

The effect of AgNO_3_ at different initial concentrations in the reaction is exhibited in Figure 2b. The highest absorption peak position of the prepared colloids was around 400 nm. It is generally believed that the formation of AgNPs includes two processes, nucleation and crystal growth [30]. These two processes existed simultaneously in the entire reaction of adding AgNO_3_ solution, but nucleation dominated during the early stage, and nuclear growth dominated later. As the concentration of silver ions in the colloid increased (0.01, 0.02, and 0.03 mol/L), more nuclei were formed during the early stage, and sufficient silver was supplied to the nuclei for growth. Therefore, the concentration of nano silver increased, and the absorption peak position was higher. By further increasing the AgNO_3_ concentration, the maximum absorbance value began to show a downward trend. We infer that the scope and ability of the co-protective agents were limited. Increasing the AgNO_3_ concentration would have resulted in frequent collisions between grains, thereby leading to the aggregation and deposition of nanoparticles. Furthermore, we also reported the pH of the solution before and after the AgNPs were synthesized in Table 1.

The pH is an important factor affecting the ionization equilibrium and electrode potential of the reaction system. Before the synthesis of AgNPs, the overall solution showed alkalinity due to the ionization of sodium hydroxide. Moreover, as the concentration of AgNO_3_ increased, the alkalinity of the reaction system decreased. It was because the hydrolysis of AgNO_3_ showed weak acidity, which neutralized part of hydroxyl ion (OH^−^). After the synthesis, the reaction solution showed acidity, indicating that OH^−^ participated in reduction of Ag^+^ to form Ag by glucose and generated acidic substances. The addition of alkali was conducive to higher reducing ability; however, it had an adverse effect on particle agglomeration [31]. According to the acid–base change of the solution, we speculated that the following reaction process might occur during the synthesis of ps-AgNPs:

2Ag^+^ + 2OH^−^ → Ag_2_O + H_2_O (2)

Ag_2_O + CH_2_OH (CHOH)_4_CHO + 2PVP → CH2OH (CHOH)_4_COOH + 2Ag(PVP)↓ (3)

### 3.2. Structural Characterization

#### 3.2.1. XRD Analysis

**Figure 3:**
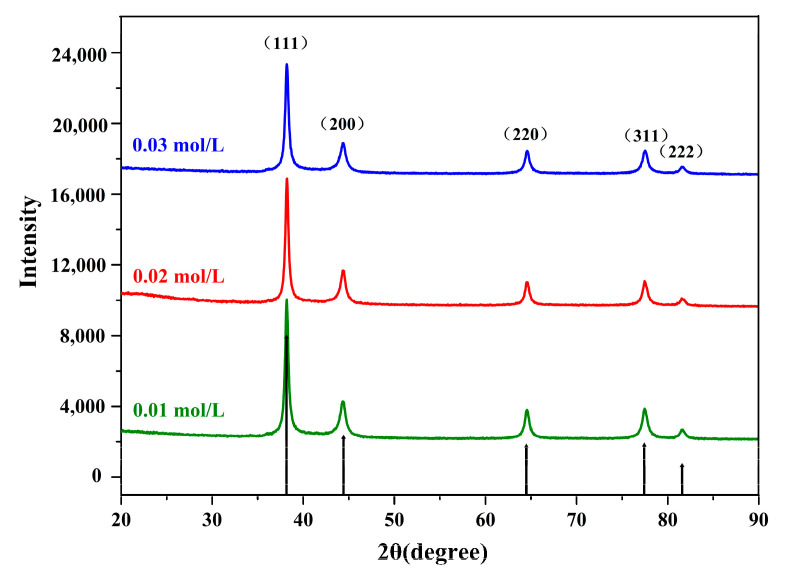
Diffractograms of synthesized polyvinylpyrrolidone (PVP) plus sodium tripolyphosphate (STPP) silver nanoparticles (ps-AgNPs), with different initial concentrations of AgNO_3_.

Figure 3 presents the ps-AgNPs spectra prepared under the initial AgNO_3_ concentrations of 0.01, 0.02, and 0.03 mol/L. Through X’ pert High Score Plus software analysis, the similarities with the standard silver crystal XRD diffraction card (01-087-0717) were 92%, 94%, and 96%, respectively (Figure S3). Moreover, the diffraction peaks of the sample were relatively clear and single, without miscellaneous peaks, thus indicating that AgNPs of a high purity and stable size were synthesized. The XRD pattern of AgNPs showed five characteristic 2θ peaks, indexing Bragg’s reflections planes (111), (200), (220), (222), and (311), which confirmed the face-centered cubic (fcc) phase of Ag. Then, based on the crystal plane corresponding to the highest peak (111), the average particle sizes in the direction of the peaks were all 32.4 nm (Tables S4–S6), as calculated by the Scherrer equation, thus proving that nanoscale elemental Ag was generated.

#### 3.2.2. FE-SEM and TEM Analysis

**Figure 4:**
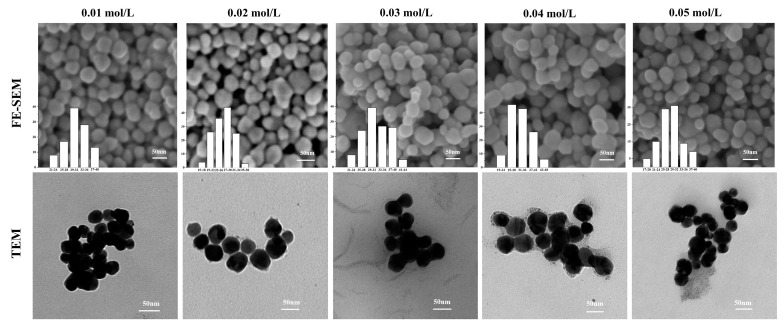
Images and histograms of polyvinylpyrrolidone (PVP) plus sodium tripolyphosphate (STPP) silver nanoparticles (ps-AgNPs) with different AgNO_3_ concentrations. FE-SEM: field emission scanning electron microscopy; TEM: transmission electron microscopy.

The images of synthesized ps-AgNPs at different equimolar concentrations (0.01, 0.02, 0.03, 0.04, and 0.05 mol/L) are shown in Figure 4. The FE-SEM analysis revealed that ps-AgNPs had a smooth spherical surface. Due to the “encapsulation” of the protective agent, the edges of the particles were lighter than the centers, causing them to be oval/elliptical in shape [32]. The characteristics of agglomeration among nanoparticles were also observed on account of their high surface tension and energy. Besides, by comparing the particle size distribution of ps-AgNPs prepared at different concentrations, we found they were 30.9 ± 4.2 nm, 26.6 ± 4.4 nm, 31.9 ± 5.0 nm, 31.8 ± 5.9 nm, and 28.2 ± 4.9 nm, which were consistent with the particle sizes estimated by XRD. Moreover, TEM provided further information regarding the morphology and size distribution of the synthesized nano silver. In this concentration range, the morphology and particle size of nano silver remained basically consistent, with the conclusions of the XRD and FE-SEM analyses. Their morphology was spheroidal, and the particle-size-variation range was stable.

**Figure 5:**
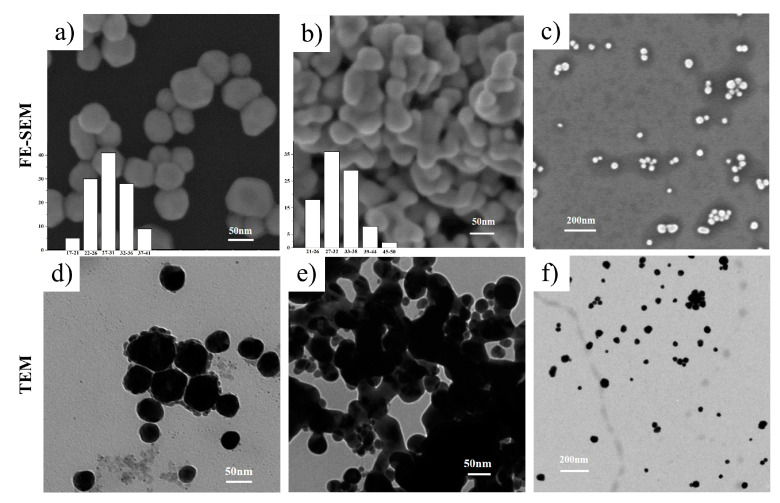
Images of (**a**,**d**) polyvinylpyrrolidone silver nanoparticles (p-AgNPs) and (**b**,**e**) sodium tripolyphosphate silver nanoparticles (s-AgNPs) at 0.02 mol/L AgNO_3_, and (**c**,**f**) images of polyvinylpyrrolidone (PVP) plus sodium tripolyphosphate (STPP) silver nanoparticles (ps-AgNPs) at 0.01 mol/L AgNO_3_ in low magnification. FE-SEM: field emission scanning electron microscopy; TEM: transmission electron microscopy.

With respect to the p-AgNPs and s-AgNPs, Figure 5 presents their FE-SEM and TEM images for clear comparison. The p-AgNPs had relatively neat edges and better dispersion. However, the morphology of the s-AgNPs was stretched to a certain extent and exhibited a short, thick rod-like shape. Moreover, their particles formed agglomerates, which implies that STPP led to the tendency of nanoparticles to stretch [13] and agglomerate. In addition, it transpired that the agglomeration force could be opened by ultrasonic vibration, given an appropriate power and time, as shown in the images under low magnification.

**Figure 6:**
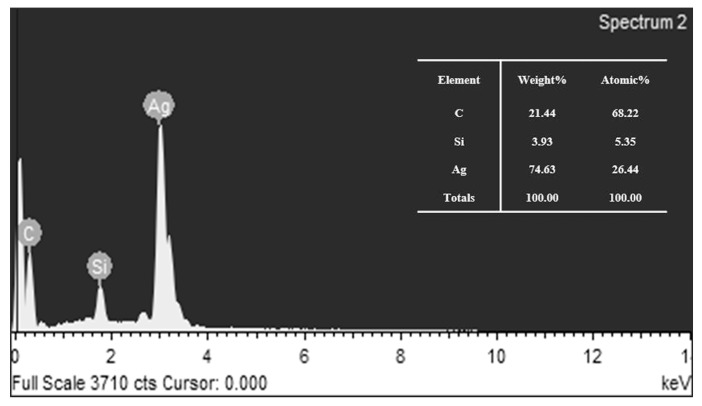
Energy dispersive X-ray (EDX) spectra of polyvinylpyrrolidone (PVP) plus sodium tripolyphosphate (STPP) silver nanoparticles (ps-AgNPs) at 0.02 mol/L AgNO_3_.

EDX spectra (Figure 6) analysis confirmed the presence of elemental silver by sharp signals around 3 keV, which is a typical range of metallic nano crystallites optical absorption band [33]. The peak observed at 1.75 keV belonged to Si, which was formed from the SEM carrier. A spurious peak between 0 and 2 keV that corresponds to C was derived from the raw materials, and C was coated onto the surface of AgNPs in the form of protective agents. No other obvious impurity peaks were detected throughout the scanning range of binding energies. Thus, it was apparent that the EDX analysis also provided evidence of high-purity AgNPs.

#### 3.2.3. Zeta Potential

**Figure 7:**
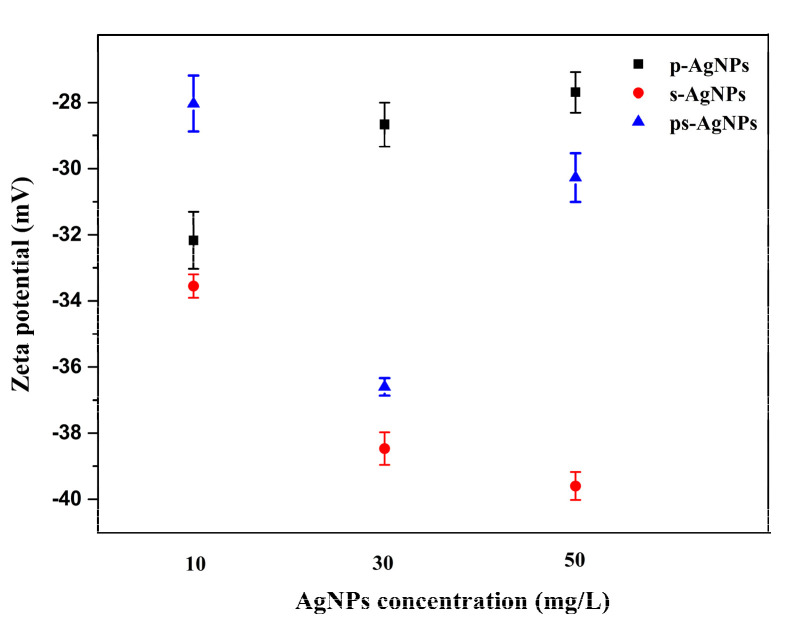
Zeta potentials of the three silver nanoparticles (AgNPs) at different concentrations. Polyvinylpyrrolidone is indicated by p-, and sodium tripolyphosphate by s-.

As shown in Figure 7, zeta potential was tested for different concentrations and types of nano silver, to determine their stability. Overall, the synthesized AgNPs were negatively charged, which kept the nanoparticles in the colloidal suspension. With increased nano silver concentration, the zeta potential of p-AgNPs decreased and that of the s-AgNPs increased. PVP is primarily used to stabilize nanoparticles by providing steric resistance, rather than by electrostatic repulsion [34]. As a result, p-AgNPs is less electronegative. The tripolyphosphate (P_3_O_10_^5−^) produced by STPP acted as a polyanion that interacts with cationic substances by electrostatic force [35]. As its concentration increased, this electrostatic repulsion became more pronounced. The zeta potential of ps-AgNPs was lower at lower concentrations, thus showing an insignificant electrostatic repulsion. When the concentration was increased, the zeta potential was centered in contrast with the other two AgNPs, thereby indicating that PVP limited the polynegative effect of P_3_O_10_^5−^. Combining the microscopy images of the three AgNPs, we observed that the aggregation state of s-AgNPs was the worst, while p-AgNPs and ps-AgNPs had better dispersibility. It was concluded that the steric hindrance of PVP played a leading role in maintaining the stability of nanoparticles.

#### 3.2.4. FTIR Analysis

**Figure 8:**
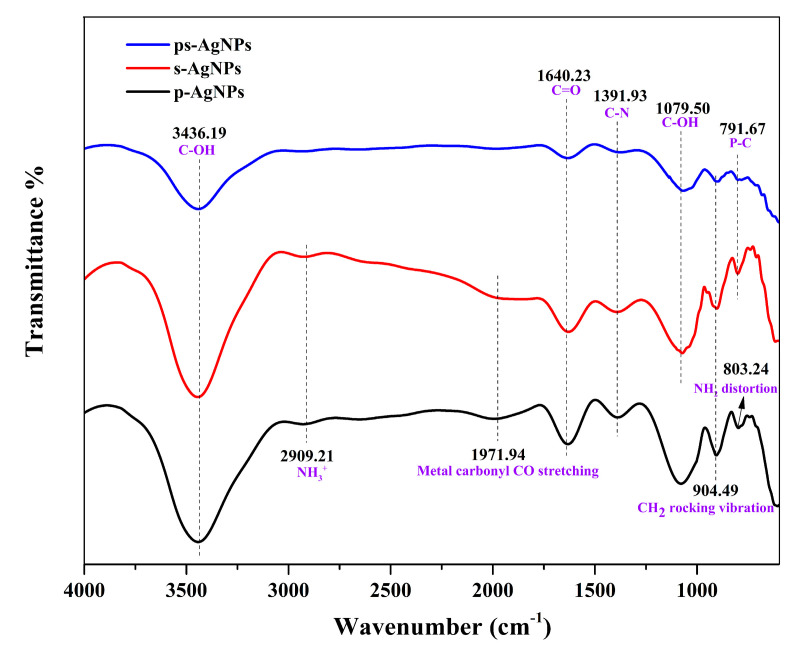
Fourier transform infrared spectroscopy (FTIR) spectra of three silver nanoparticles (AgNPs). Polyvinylpyrrolidone is indicated by p-, and sodium tripolyphosphate by s-.

The surface functional groups of three AgNPs were determined by FTIR, as shown in Figure 8. In comparison with the frequency table of organic compound group vibration [36], the results of the peaks in the same position have been marked. The analysis indicated that the spectra of p-AgNPs, s-AgNPs and ps-AgNPs exhibited the same bands at 3346.19, 1640.23, 1391.93, 1079.50, and 904.49 cm^−1^. By comparing the expansion of the functional group NH3+ that corresponded to the diffused absorption band at 2909.21 cm^−1^, we speculate that NO_3_^−^ could be reduced to NH^3+^ by glucose only under the action of PVP and STPP alone. However, when both of the protective agents were present, this reaction rarely occurred, and the reduction of Ag^+^ by glucose was merely carried out. On the other hand, the metal carbonyl CO stretching at 1971.94 cm^−1^ indicates that the aldehyde group (CHO) of glucose had been broken and formed the metal carbonyl with the transition metal Ag. Similarly, this reaction did not occur under the coupling of PVP and STPP. It is worth noting that the peaks at 2909.21 and 1971.94 cm^−1^ were weak, indicating a small reaction intensity. Additionally, the distorted vibration of the functional group NH2 at 803.24 cm^−1^ also supported the aforementioned conjecture.

### 3.3. Formation Mechanism

The formation of nanoparticles mainly includes three stages: reduction of ions, grouping of nanoparticles, and subsequent growth of nanoparticles. Moreover, the reduction process of metal ions is affected by multiple factors, including the reaction conditions (e.g., temperature, pH, and reaction time) of the mixture system, electrochemical changes of metal ions, and properties of the protective agents [37].

The following are formation mechanisms for the synthesis of ps-AgNPs with a stable particle size. Firstly, during the reduction of Ag^+^ to silver, STPP as a protective agent can dissociate into P_3_O_10_^5−^, which would have combined with Ag^+^ to form silver triphosphate (Ag_5_P_3_O_10_). As a consequence of the limited solubility of Ag_5_P_3_O_10_ in the solution, the concentration of Ag^+^ is maintained at a stable level, which prevents the uneven particle size distribution caused by an excessive reduction rate and stabilizes the particle size. At the same time, the addition of PVP also promotes the stabilization of the colloid and prevents agglomeration among particles. This is mainly because the polar groups in its structural unit contain N and O atoms, both of which have lone pair electrons such that they can provide electron clouds for empty *sp* hybrid orbitals of Ag^+^ to form coordination bonds [38]. That is, the *sp* orbits are hybridized to form a silver ion complex. Moreover, the linear PVP can continue to adsorb on the surface of colloidal particles after the reduction of silver. The protective layer formed at the solid–liquid interface can not only effectively reduce the surface energy, but also uniformly disperse AgNPs in the liquid-phase system [39]. Meanwhile, the linear induction of P_3_O_10_^5−^ during the growth of silver nuclei is prevented, and the agglomeration problem is alleviated. In addition, the coupling of PVP and STPP as protective agents may also limit the progress of other redox reactions.

**Figure 9:**
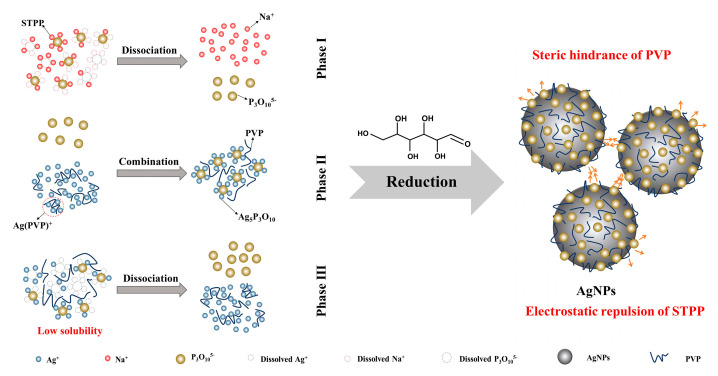
Formation process of polyvinylpyrrolidone plus sodium tripolyphosphate silver nanoparticles (ps-AgNPs). PVP: polyvinylpyrrolidone; STPP: sodium tripolyphosphate.

Nevertheless, we observed that, regardless of the type of protective agent that was used to prepare the nano silver, different degrees of aggregation would occur after a period of time. This indicates that the dispersion effect of the protective agent would weaken with time. As a result of the high specific surface area and surface energy, the nano silver enters a thermodynamically unstable state, and the particles easily fuse with each other, in order to reunite [40]. Furthermore, the agglomeration force can still be opened by ultrasonic dispersion. The synthesis process of the ps-AgNPs is illustrated in Figure 9.

### 3.4. Anti-Algal Activity of AgNPs

**Figure 10:**
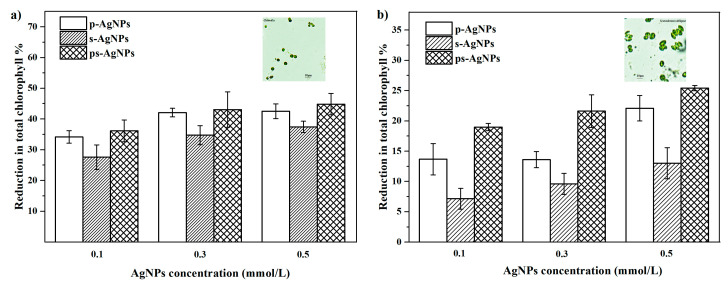
Reduction in total chlorophyll in algae at varying concentrations and types of silver nanoparticles (AgNPs). (**a**) *Chlorella*. (**b**) *Scenedesmus obliquus*. Polyvinylpyrrolidone is indicated by p-, and sodium tripolyphosphate by s-.

The effect of AgNPs on the chlorophyll content was measured, in order to observe the sensitivity of two algae toward their toxicity. Here, we compared the anti-algal toxicity with p-AgNPs, s-AgNPs, and ps-AgNPs (Figure 10).

The total chlorophyll content in both the algal species (*Chlorella* and *Scenedesmus obliquus*) exhibited a concentration-dependent decrease following exposure to the three AgNPs at different concentrations (0.1, 0.3, and 0.5 mmol/L). The percentage of the total chlorophyll reduction increased with exposure to the AgNPs. On the third day of *Scenedesmus obliquus* cultivation, the chlorophyll content decreased significantly, by 19.0%, 21.6%, and 25.4%, in response to the applied ps-AgNPs at concentrations of 0.1, 0.3, and 0.5 mmol/L. Similarly, the reduction in the chlorophyll content was more pronounced, exhibiting declines of approximately 36.2%, 43.1%, and 44.8%, at the corresponding doses of ps-AgNPs. The decreased chlorophyll content that was caused by the silver toxicity, which was more evident in *Chlorella* than in *Scenedesmus obliquus*, thus indicating a greater sensitivity of *Chlorella* against nano silver toxicity. Additionally, the chlorophyll content of s-AgNPs decreased the least; that is, the effect of s-AgNPs on photosynthetic pigments of algae was the weakest. Therefore, the s-AgNPs presented a lower algae resistance than the other two AgNPs. Nano silver can be introduced to accumulate slightly due to the presence of electrolytes in the BG11 medium [41]. As a result, the s-AgNPs, which carried the most negative charges, were even less dispersed. The dispersion state of nano silver has an important impact on its bioavailability and environmental behavior. It is generally believed that nanoparticles can enter cells through holes in the cell wall [42]. Therefore, the severely agglomerated s-AgNPs showed a poor anti-algal toxicity. At the same time, nanomaterials are more stable through electrostatic repulsion and steric hindrance [43], which enhance the inhibition effect of the ps-AgNPs on the algae photosynthetic pigment stability, thereby showing a better algae-suppression effect.

## 4. Conclusions

In summary, we used a new approach for the synthesis of AgNPs with a stable size and better dispersibility, by combining PVP and STPP, and then we investigated the coupling mechanism and tested its anti-algal potential. During the synthesis process, the AgNPs prepared by the synergistic protection of PVP and STPP displayed a superior state. The structures analysis demonstrated that the synthesized pure AgNPs was of a stable sized (~30 nm in diameter). The coupling mechanism of the co-protective agents was proposed from the perspective of colloidal interface and potential. The low-solubility Ag_5_P_3_O_10_ formed from STPP and the steric hindrance of PVP jointly limited the reduction rate of Ag^+^, preventing the uneven particle size distribution caused by the excessive reduction rate and stabilizing the particle size. Meanwhile, PVP limited the polynegative effect of P_3_O_10_^5−^ and prevented the linear induction of P_3_O_10_^5−^ during the growth of silver nuclei. Ultimately, the AgNPs synthesized with co-protective agents exhibited the potential inhibitory ability in algal-resistance experiments against *Chlorella* and *Scenedesmus obliquus*. This study provides a basic theory for the synthesis of AgNPs induced by various factors in the natural environment and a scientific reference for the risk assessment of water environment. For future studies, the co-effects of multiple protection mechanisms on prepared materials can be further explored, to investigate whether the anti-algal potential of nano silver is stable and durable.

## Figures and Tables

**Table 1:** The pH of the solution before and after the ps-AgNPs were synthesized with different initial concentrations of AgNO_3_.

The Concentration of AgNO_3_ (mol/L)	0.01	0.02	0.03	0.04	0.05
pH before synthesis	10.58	10.35	10.17	10.22	10.14
pH after synthesis	5.82	5.44	4.74	4.67	4.83

## Supplementary Materials

The following are available online at https://www.mdpi.com/2079-4991/10/6/1042/s1, Figure S1: Growth curves of *Chlorella* and *Scenedesmus obliquus*, Figure S2: Linear relationships between algal concentration and absorbance, Figure S3: Matching result of X’ pert High Score Plus software in XRD analysis (a) ps-AgNPs with 0.01 mol/L of AgNO_3_. (b) ps-AgNPs with 0.02 mol/L of AgNO_3_. (c) ps-AgNPs with 0.03 mol/L of AgNO_3_, Table S1: Parameters for preparing ps-AgNPs, Table S2: Parameters for preparing p-AgNPs, Table S3: Parameters for preparing s-AgNPs, Table S4: XRD data of ps-AgNPs with 0.01 mol/L of AgNO_3_, Table S5: XRD data of ps-AgNPs with 0.02 mol/L of AgNO_3_, Table S6: XRD data of ps-AgNPs with 0.03 mol/L of AgNO_3_.
