# Supplementary material for: Dispersibility and Size Control of Silver Nanoparticles with Anti-Algal Potential Based on Coupling Effects of Polyvinylpyrrolidone and Sodium Tripolyphosphate
Source: Nanomaterials (Basel). 2020 May 29;10(6):1042. doi: 10.3390/nano10061042 (PMC7352764; doi:10.3390/nano10061042)
Supplement: Supplementary file 1 [file nanomaterials-10-01042-s001.pdf]

Supplementary Material

Dispersibility and Size Control of Silver Nanoparticles with Anti-Algal Potential Based on Coupling Effects of Polyvinylpyrrolidone and Sodium Tripolyphosphate

Mingshuai Wang, Haibo Li *, Yinghua Li *, Fan Mo, Zhe Li, Rui Chai and Hongxuan Wang

School of Resources and Civil Engineering, Northeastern University, Shenyang 110819, China; wms1995@126.com (M.W.); moliangming4312975@163.com (F.M.); lizhe1824@163.com (Z.L.); chairuicici@126.com (R.C.); 18245706557@163.com (H.W.)

* Correspondence: lihaibo@mail.neu.edu.cn (H.L.); liyinghua@mail.neu.edu.cn (Y.L.)

Received: 10 May 2020; Accepted: 27 May 2020; Published: 29 May 2020

Table S1. Parameters for preparing ps-AgNPs.

Materials	Silver nitrate	PVP	STPP	Glucose	Sodium hydroxide
Dosage (mL)	25	12.5	6.25	15	40
	0.01	0.1	0.05	0.1	0.05
	0.02	0.1	0.05	0.1	0.05
Concentration (mol/L)	0.03	0.1	0.05	0.1	0.05
	0.04	0.1	0.05	0.1	0.05
	0.05	0.1	0.05	0.1	0.05

Table S2. Parameters for preparing p-AgNPs.

Materials	Silver nitrate	PVP	Glucose	Sodium hydroxide
Dosage (mL)	25	12.5	15	40
Concentration (mg/L)	0.02	0.1	0.1	0.05

Table S3. Parameters for preparing s-AgNPs.

Materials	Silver nitrate	STPP	Glucose	Sodium hydroxide
Dosage (mL)	25	6.25	15	40
Concentration (mg/L)	0.02	0.05	0.1	0.05

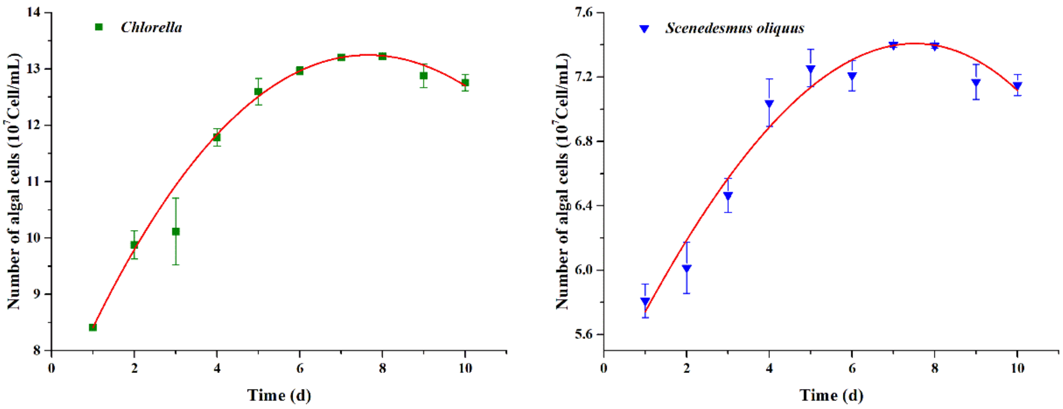

Figure S1. Growth curves of *Chlorella* and *Scenedesmus obliquus*.

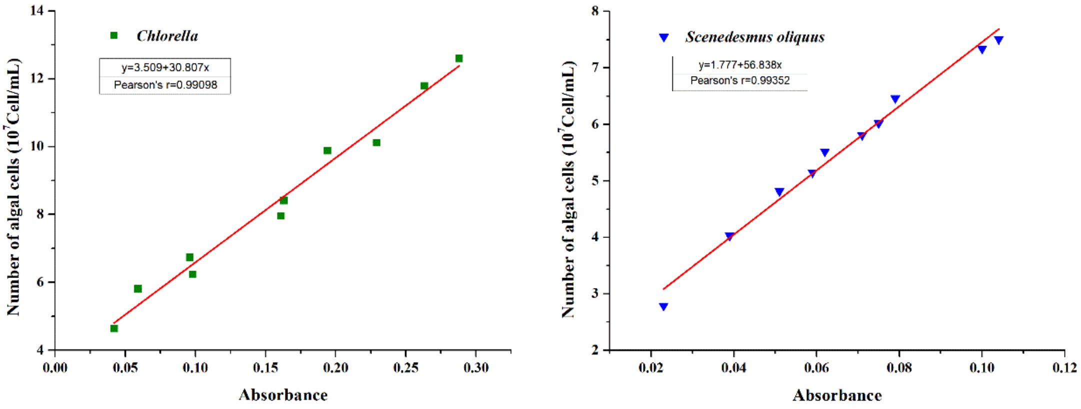

Figure S2. Linear relationships between algal concentration and absorbance.

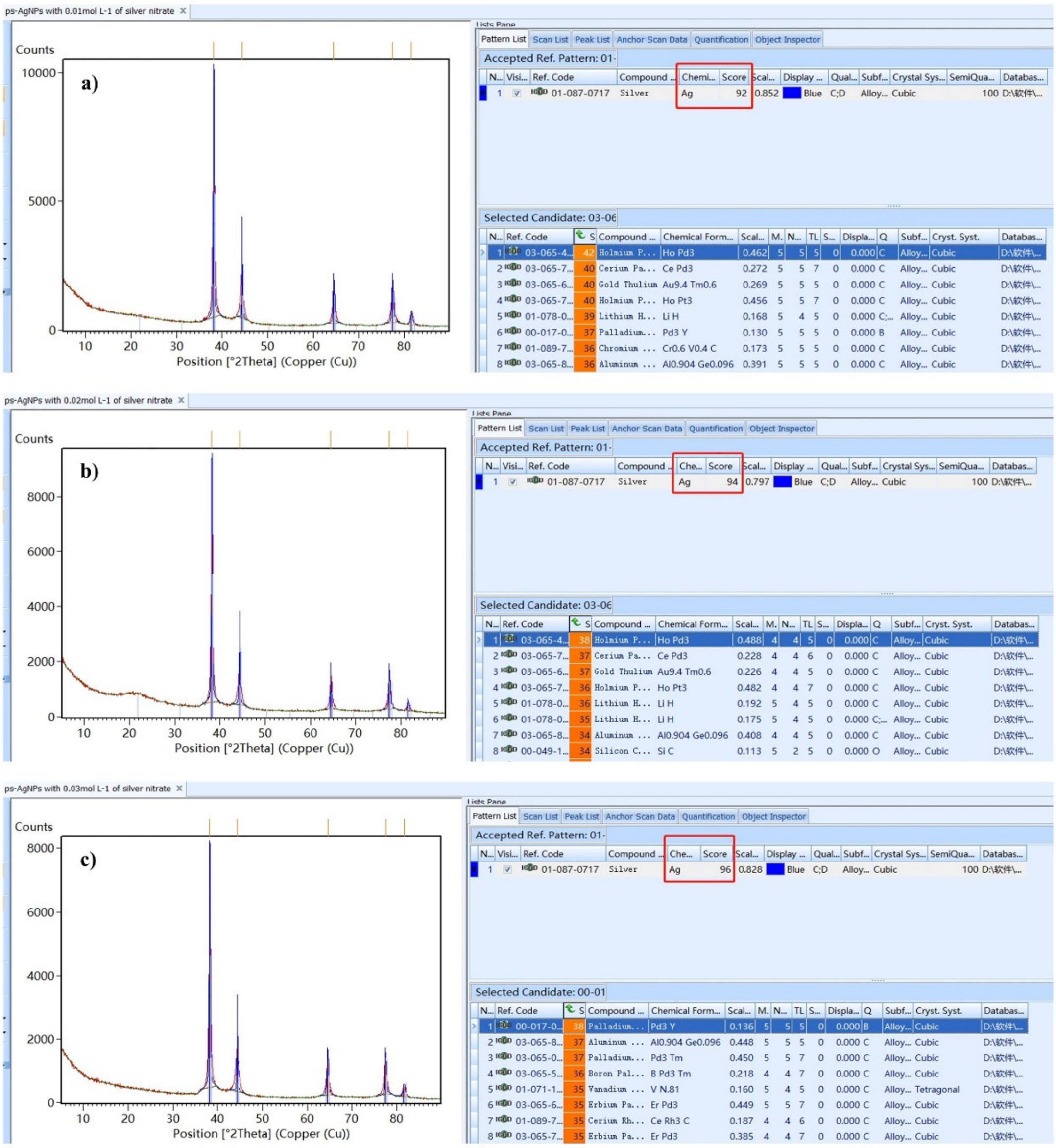

Figure S3. Matching result of X' pert High Score Plus software in XRD analysis. a) ps-AgNPs with 0.01 mol/L of $AgNO_3$. b) ps-AgNPs with 0.02 mol/L of $AgNO_3$. c) ps-AgNPs with 0.03 mol/L of $AgNO_3$.

Table S4. XRD data of ps-AgNPs with 0.01 mol/L of $AgNO_3$.

#	(hkl)	2θ (deg)	FWHM (deg)	d (Å)	D(nm)
1	(111)	38.1597	0.2598	2.3584	32.37

2	(200)	44.4250	0.1948	2.0393	44.07
3	(220)	64.4888	0.2922	1.4450	32.15
4	(311)	77.4215	0.3247	1.2327	31.36
5	(222)	81.5864	0.4546	1.1800	23.09

Table S5. XRD data of ps-AgNPs with 0.02 mol/L of $AgNO_3$.

#	(hkl)	2θ (deg)	FWHM (deg)	d (Å)	D(nm)
1	(111)	38.1954	0.2598	2.3563	32.37
2	(200)	44.3281	0.2922	2.0435	29.37
3	(220)	64.4862	0.1624	1.4450	57.85
4	(311)	77.4608	0.2922	1.2322	34.86
5	(222)	81.5469	0.3247	1.1805	32.31

Table S6. XRD data of ps-AgNPs with 0.03 mol/L of $AgNO_3$.

#	(hkl)	2θ (deg)	FWHM (deg)	d (Å)	D(nm)
1	(111)	38.1708	0.2598	2.3578	32.37
2	(200)	44.3684	0.2273	2.0418	37.76
3	(220)	64.5459	0.3247	1.4438	28.95
4	(311)	77.5009	0.2922	1.2317	34.87
5	(222)	81.6329	0.4546	1.1795	23.09
